# Autism screening at 18 months of age: a comparison of the Q-CHAT-10 and M-CHAT screeners

**DOI:** 10.1186/s13229-021-00480-4

**Published:** 2022-01-03

**Authors:** Raymond Sturner, Barbara Howard, Paul Bergmann, Shana Attar, Lydia Stewart-Artz, Kerry Bet, Carrie Allison, Simon Baron-Cohen

**Affiliations:** 1 Pediatrics, Johns Hopkins School of Medicine, Baltimore, USA; 2 Center for Promotion of Child Development Through Primary Care, Baltimore, MD USA; 3 CHADIS, Inc., 6017 Altamont Place, Baltimore, MD USA; 4 Foresight Logic, Inc., St. Paul, MN USA; 5 Autism Research Centre, Department of Psychiatry, University of Cambridge, Cambridge, UK; 6 University of Washington, Seattle, WA USA

**Keywords:** Autism screening, Developmental screening, M-CHAT, Q-CHAT

## Abstract

**Background:**

Autism screening is recommended at 18- and 24-month pediatric well visits. The Modified Checklist for Autism in Toddlers—Revised (M-CHAT-R) authors recommend a follow-up interview (M-CHAT-R/F) when positive. M-CHAT-R/F may be less accurate for 18-month-olds than 24-month-olds and accuracy for identification prior to two years is not known in samples that include children screening negative. Since autism symptoms may emerge gradually, ordinally scoring items based on the full range of response options, such as in the 10-item version of the Quantitative Checklist for Autism in Toddlers (Q-CHAT-10), might better capture autism signs than the dichotomous (i.e., yes/no) items in M-CHAT-R or the pass/fail scoring of Q-CHAT-10 items. The aims of this study were to determine and compare the accuracy of the M-CHAT-R/F and the Q-CHAT-10 and to describe the accuracy of the ordinally scored Q-CHAT-10 (Q-CHAT-10-O) for predicting autism in a sample of children who were screened at 18 months.

**Methods:**

This is a community pediatrics validation study with screen positive (*n* = 167) and age- and practice-matched screen negative children (*n* = 241) recruited for diagnostic evaluations completed prior to 2 years old.

Clinical diagnosis of autism was based on results of in-person diagnostic autism evaluations by research reliable testers blind to screening results and using the Autism Diagnostic Observation Schedule—Second Edition (ADOS-2) Toddler Module and Mullen Scales of Early Learning (MSEL) per standard guidelines.

**Results:**

While the M-CHAT-R/F had higher specificity and PPV compared to M-CHAT-R, Q-CHAT-10-O showed higher sensitivity than M-CHAT-R/F and Q-CHAT-10.

**Limitations:**

Many parents declined participation and the sample is over-represented by higher educated parents. Results cannot be extended to older ages.

**Conclusions:**

Limitations of the currently recommended two-stage M-CHAT-R/F at the 18-month visit include low sensitivity with minimal balancing benefit of improved PPV from the follow-up interview. Ordinal, rather than dichotomous, scoring of autism screening items appears to be beneficial at this age. The Q-CHAT-10-O with ordinal scoring shows advantages to M-CHAT-R/F with half the number of items, no requirement for a follow-up interview, and improved sensitivity. Yet, Q-CHAT-10-O sensitivity is less than M-CHAT-R (without follow-up) and specificity is less than the two-stage procedure. Such limitations are consistent with recognition that screening needs to recur beyond this age.

**Supplementary Information:**

The online version contains supplementary material available at 10.1186/s13229-021-00480-4.

## Background

Autism spectrum disorder (henceforth autism) is a prevalent and life-long condition, with a rate of 1 in 54 [1] by 8 years of age. A strong association between early evidence-based intervention with improved long-term outcomes for children with autism is one rationale for the recommendation by the American Academy of Pediatrics (AAP) and the Centers for Disease Control and Prevention (CDC) for screening of autism in all children at 18 and 24 months [2–7]. However, the 2015 US Preventive Services Task Force (USPSTF) asserted that additional data are needed, in part due to a lack of adequate validation of the recommended tools in community samples [8].

The Checklist for Autism in Toddlers (CHAT), one of the first validated autism screening tests, showed initial promise for screening at 18-months with a high concurrent positive predictive value (PPV) [9]. However, at a 6-year follow-up, the 18-month CHAT had only identified 38% of children with an autism diagnosis [10]. The low sensitivity of the CHAT, and a desire to eliminate its child observation items, led to modifications of the screen, e.g., the Modified CHAT (M-CHAT) [11], that also added parent report items. Another modification, the Quantitative CHAT (Q-CHAT) [12], changed the dichotomous responses (yes/no) of the CHAT and M-CHAT to ordinal responses (how much/often), acknowledging autistic traits lie on a dimension [13].

While the M-CHAT is the most widely used autism screening test, it may not exceed the CHAT in long-term sensitivity, in part due to children that are not even detectable by diagnostic testing until a later age [14]. The revised M-CHAT (M-CHAT-R) authors now highly recommend use of a standardized follow-up clinician-administered interview for most positive screens [15]. Of note, during validation studies, the follow-up interview was conducted by telephone as part of a “two-stage screener” process known as the M-CHAT-R/F, which increased PPV from 0.14 to 0.48 in a sample of M-CHAT-R screen-positive children at 18- and 24-month well-child visits [16]. In a high-risk sample of siblings of autistic individuals, the M-CHAT-R/F appeared to have good sensitivity (0.78) at 18 months. However, nearly one-fifth of those screening negative on the M-CHAT-R/F later were found to have an autism diagnosis [14]. However, the follow-up interview has an extremely low rate of utilization in primary care settings [17]. Even with the follow-up interview, the PPV was lower in younger toddlers compared to older toddlers in one community sample (0.28 vs. 0.61, respectively), and similarly lower in another (0.36 vs. 0.69, respectively) [18, 19]. Results of samples with a high prevalence of autism, such as siblings of autistic children, cannot be generalized to typical community samples. Also, neither the M-CHAT-R nor the 10-item version of the Q-CHAT (Q-CHAT-10) has been studied in a representative community population of 18-month-olds with validation testing that includes both screen negatives and positives as needed to better estimate sensitivity. Furthermore, the different item response approaches of the ordinal version of the Q-CHAT-10 (Q-CHAT-10-O) and the dichotomous M-CHAT-R have never been directly compared.

Studies show that when the full cohort of toddlers screened by the M-CHAT is followed for several years, M-CHAT sensitivity and PPV are lower than in the concurrent validation studies of M-CHAT positive children because of the later emergence of autistic symptoms making diagnosis possible. In these follow-up studies predictive indices are lowest for the youngest toddlers. For example, a follow-up study in Norway showed that a positive M-CHAT (without follow-up) at 18 months identified only 34% of children with an autism diagnosis by 9 years old [20]. A recent report of screening with the M-CHAT at both the 18- and 24-month well-child visits, whose medical records were reviewed for autism diagnoses as outcomes at 4 to 8 years of age, reported a similar sensitivity of 0.35 for 18-month screening; lower than a sensitivity of 0.49 at 24 months [21]. A sensitivity of 0.33 was found in a similar cohort follow-up reported as a combined 18- and 24-month sample [22]. In 2019, Guthrie, et al. found for the 41.2% of children whose score triggered the follow-up portion of the M-CHAT-R/F, the PPV was also higher at the 24-month visit than when the same child was screened at 18 months (0.25 and 0.18, respectively) [21].

An obstacle to estimates of prediction of autism diagnoses made years later is that some children may not have had any clinical manifestations at the earlier age and thus negative screens were ambiguous. In addition, a meta-analysis of reports shows that an average of 32% of toddlers, with an eventual diagnosis of autism, look typical at 18 months and then are reported at a later age to have regressed between 18- and 24-months [23]—one reason the AAP recommends rescreening at 24 months [6]. Additionally, data from prospective studies of high-risk infant siblings reveal that only 18% of children diagnosed with autism at 36 months were given that diagnosis at 18 months of age despite use of comprehensive diagnostic assessments.^24^ Prevalence is also reported as 30% higher at ages 8–12 years than at 3–7 years [25]. Children identified later with autism tend to have milder symptoms and higher cognitive functioning [26].

Strategies that are age-relevant and capture the natural emergence of autism are needed to address the screening challenges at 18 months. One approach may be sum scoring of ordinal scaled item responses such as those in the Q-CHAT [12]. The Q-CHAT-10 is particularly well suited for primary care because of its brevity, and reported sensitivity of 0.91 and specificity 0.89 in a case comparison study [27]. However, since data from community primary care populations are lacking, we cannot consider this to be a true estimate of sensitivity. Also, while the Q-CHAT-10 uses a five-point frequency response, its standard scoring instructions utilize a pass/fail cutpoint rather than ordinal scoring based on the full-scale range of the items. In this study, we compare the predictive utility of the M-CHAT-R, the M-CHAT-R/F, and the Q-CHAT-10 in a community sample that includes both toddlers who screen positive and screen negative on initial screening measures. An additional aim of this study was to compare the Q-CHAT-10 with its original pass/fail scoring to an experimental ordinal scoring version we term Q-CHAT-10-O to better understand the contribution of ordinal scoring to accurate 18-month screening.

## Methods

### Sampling and screening procedures

**Fig. 1:**
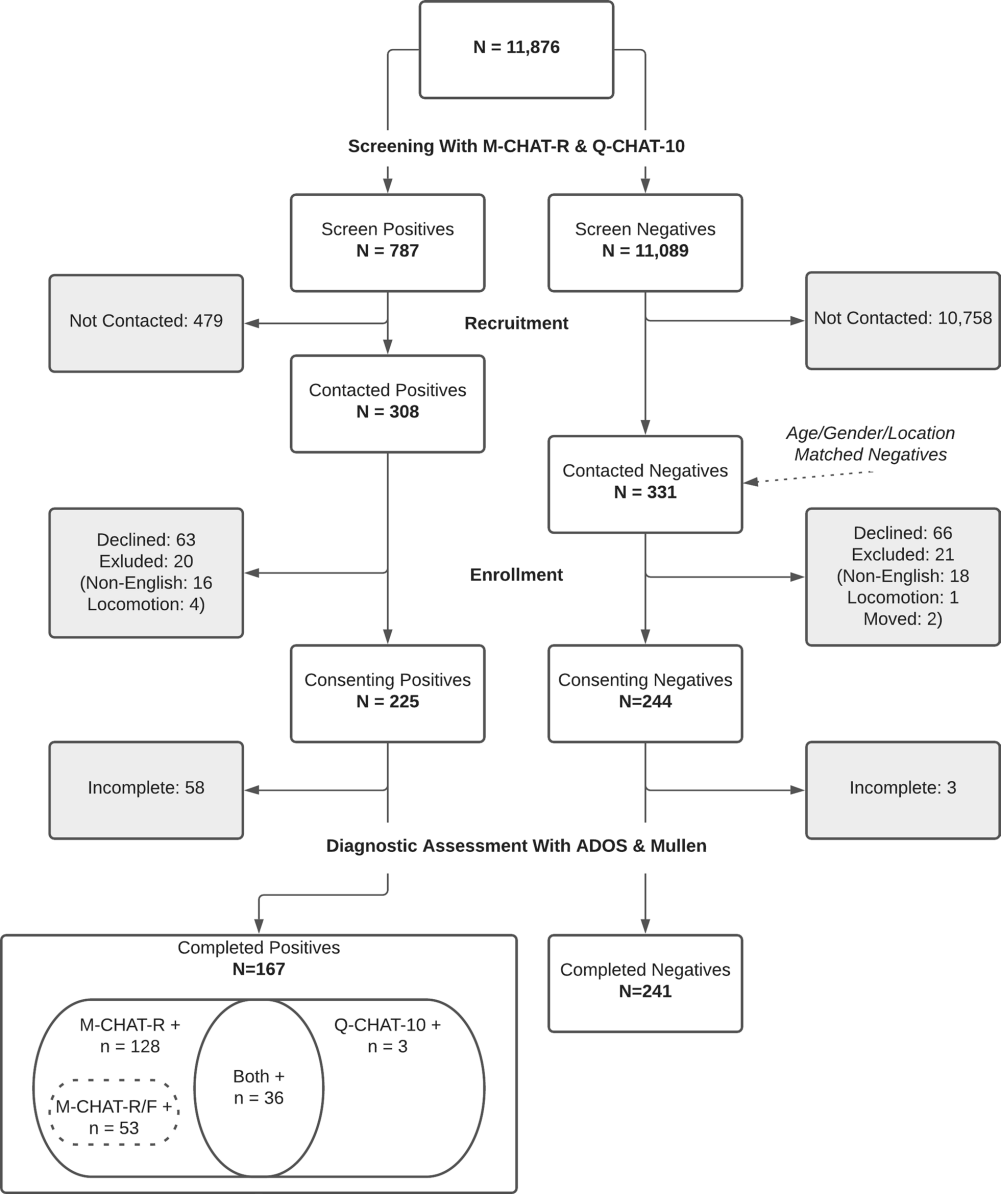
Study sample flow

Parents completed the M-CHAT-R before 18-month pediatric visits (16–20 months) via an online clinical process support system called CHADIS [28, 29]. M-CHAT-R positive screens prompted completion of M-CHAT-R/F by the PCP during or after the visit by phone, or by a research assistant using online prompts in CHADIS via a previously validated method [30]. The follow-up interview was completed for all M-CHAT-R positive parent reports except 23 which were not initiated (*n* = 17) or not completed (*n* = 6). In addition, parents completed the Q-CHAT-10 and Ages & Stages Questionnaires—Third Edition (ASQ-3) [31]. The order of presentation of Q-CHAT-10 and M-CHAT-R to parents alternated with Q-CHAT-10 being administered before M-CHAT-R one month and the reverse the following month. A total of 11,876 parents of children age 16–20 months from pediatric offices already using the CHADIS system in Maryland, Massachusetts, and North Carolina completed the M-CHAT-R and Q-CHAT-10 screens. These locations were chosen from a large national network of CHADIS users because of availability of home visiting diagnostic testers from the research team. The offices responded to a request for participation, and some of the offices received discounts in the use of the system. Pre-visit developmental and autism screening were routine in these offices and not based on any at risk estimation. Many of the pediatricians participated in web-based quality improvement activities approved by the American Board of Pediatrics for Maintenance of Certification professional credits (MOC-4) aimed at optimizing screening of all children. However, denominator data on numbers of well child visits were not available to document percentages of children whose parents may not have ever registered in the online system to complete screens. This study employed the version of the Q-CHAT-10 that has been recommended for clinical use by its authors. This version included pictures illustrating each of the items. Of 787 children with any positive screen result (Q-CHAT-10 or M-CHAT-R, even if follow-up was negative), 308 respective parents were contacted by phone or email for enrollment. Gender and age (within one month)-matched controls with both screens negative (*n* = 331) were then successfully contacted from the same practice or a practice with similar demographics in the same area. Children were excluded if their parents reported that they were exposed to English at home less than 50% of the time or if they were not yet walking or scooting as required to complete the ADOS-2 Toddler Module [32] for autism diagnostic testing. Figure 1 presents the sample flow from screening to formal assessment and diagnosis.

The study enrolled 469 children and lost 61 to attrition. The final sample includes 408 children with available data on key items and final autism case status determination. A total of 164 children were screen positives on M-CHAT-R (including 53 M-CHAT-R/F positives), and 39 were Q-CHAT-10 positive (of which 36 were also M-CHAT-R positive). There are a total of 241 who screened negative on both the M-CHAT-R and Q-CHAT-10. The initial screening component was deemed exempt by the IRB; parents of recruited children provided written consent and received a participation fee of $200 without charge for the evaluation.

## Measures

### Screens

*Modified Checklist for Autism in Toddlers, Revised With Follow-Up Interview (M-CHAT-R/F*) [11, 15, 16]. The M-CHAT-R is a parent-report checklist with 20 yes/no response options. A follow-up interview (M-CHAT-R/F) is recommended for a positive M-CHAT-R screen when 3–7 items were failed. M-CHAT-R/F has been reported to have a PPV of 0.48 in a community sample of both 18- and 24-month visits assessing only screen positive children [15].

*Q-CHAT-10 and Q-CHAT-10-O.* Quantitative Checklist for Autism in Toddlers (Q-CHAT) was developed to provide a range of response options for autism symptoms in toddlers which included 25 items [12]. A 10-item version (Q-CHAT-10) tested retrospectively on toddlers with an autism diagnosis compared to unselected toddlers reported sensitivity 0.91 and specificity 0.89 [27]. While the original Q-CHAT uses 5-point Likert scales of quantity or frequency, each item is still scored as a binary pass/fail.

### Diagnostic assessments

*Autism Diagnostic Observation Schedule, 2nd Ed. (ADOS-2) Toddler Module* [32]*.* The ADOS, a semi-structured behavior observation assessment of social and communication skills, was modified to assure accuracy for toddlers (ADOS-2 Toddler Module). The recommended score cutpoint for 12–21-month-olds yielded 0.91 sensitivity and 0.91 specificity for autism [32].

*Mullen Scales of Early Learning (MSEL)* [33]*.* MSEL is a standardized developmental test for children 3–69 months. An Early Learning Composite (ELC) is generated using scores from 4 of 5 subscales (Visual Reception, Expressive Language, Receptive Language and Fine Motor), providing a developmental quotient, which is used to determine the expected levels of communicative and social functioning.

### Diagnostic procedures

All children completed in-person diagnostic autism evaluations using the ADOS-2 Toddler Module [32] and Mullen Scales of Early Learning (MSEL) [33]. Diagnostic testers were all experienced autism evaluators; three were certified as research reliable on the toddler module prior to the evaluations and one attained reliability through video review by a certified research reliable tester before finalized scoring. All diagnostic testers were blinded to screening results.

Results of the ADOS-2 and information from the MSEL, along with parental history focused on a review of current functioning*,* informed a clinical judgment of whether a child met criteria for autism based on the Diagnostic and Statistical Manual of Mental Disorders-5 (DSM-5) [34, 35]. Developmental disorder was defined by the typical criteria for early intervention services (score > 1-1/2 SD below the mean on two or more subscales or > 2 SD on a single subscale of the MSEL) [36].

## Results

Respondents were primary caregivers, almost all mothers who tended to be well educated and privately insured (See Tables 1 and 2).

**Table 1:** Patient demographics

	*n*	%
Screening age (months)—[mean (SD)]	18.02 (0.53)	
Diagnosis age (months)—[mean (SD)]	20.49 (1.86)	
Diagnosis age (months) ASD Screen positive—[mean (SD)]	19.98 (1.50)	
Diagnosis age (months) ASD Screen negative—[mean (SD)]	20.59 (1.91)	
*Sex*		
Female	114	27.94%
Male	294	72.06%
*Race*		
Asian	19	4.71%
Black	39	9.68%
White	281	69.73%
Multiple	58	14.39%
Unknown	6	1.49%
*Hispanic or Latino*		
No	375	93.05%
Yes	27	6.70%
*Private Payer*		
Yes	346	85.86%
*Public payer*		
Yes	48	11.76%
*Other payer*		
Yes	50	12.25%
*Household income*		
< $50,000	18	6.57%
$50,000—$150,000	132	48.18%
> = $150,000	124	45.26%

**Table 2:** Respondent demographics

	*n*	%
*Relationship to child*		
Mother	374	91.67%
Father	31	7.60%
Other primary	3	0.74%
Age—[mean (SD)]	34.27 (4.34)	
*Marital status*		
Married	368	91.32%
Separated	4	0.99%
Widowed	1	0.25%
Never married	12	2.98%
Living with partner	15	3.72%
*Race*		
Asian	29	7.11%
Black	37	9.07%
White	314	76.96%
Multiple	21	5.15%
Unknown/missing	7	1.72%
*Hispanic or latino*		
No	379	94.04%
Yes	23	5.71%
*Bachelor degree*		
No	75	18.61%
Yes	328	81.39%
*Household income*		
< $50,000	18	6.57%
$50,000—$150,000	132	48.18%
> = $150,000	124	45.26%

ADOS-2 & MSEL scores are presented in Table 3. Mean t-scores on all scales of the MSEL for children determined to have autism diagnoses were significantly lower than for those without a diagnosis (See Additional file 1: Table S1).

**Table 3:** Diagnostic score results

	*n*	Mean	SD	Min	Max
*ADOS scores (overall)*					
Social affect total	406	5.0	4.9	0	20
Restricted/repetitive behavior total	405	1.7	1.9	0	8
Overall total (SA + RRB)	405	6.6	6.1	0	24
Range of concerns	401	1.4	0.8	1	3
*ADOS scores (ASD negative)*					
Social affect total	344	3.5	3.2	0	14
Restricted/repetitive behavior total	343	1.2	1.5	0	6
Overall total (SA + RRB)	343	4.7	4.0	0	17
*ADOS scores (ASD positive)*					
Social affect total	62	13.4	3.6	6	20
Restricted/repetitive behavior total	62	4.1	1.9	1	8
Overall total (SA + RRB)	62	17.5	3.7	9	24
*MSEL T-scores*					
Gross motor	405	50.8	9.6	20	80
Visual reception	404	54.5	12.0	20	80
Fine motor	405	50.6	9.2	20	80
Receptive language	404	50.3	9.2	20	80
Expressive language	405	45.9	13.7	20	80

Similarly, mean scores on the MSEL of children screening positive on any of the autism screens were significantly lower on all scales than for those screening negative (See Additional file 1: Tables S2-S5).

The demographic profile of children diagnosed with autism differed from those who were not found to have autism by being more likely to have a family income < $50,000 and less likely to have private insurance (See Additional file 2: Table S1). The adult respondents (almost all parents) for children who were found to have an autism diagnosis were less likely to be married, and more likely to have a household income of < $50,000 and not have a college degree (See Additional file 2: Table S6). There were no overall differences in being diagnosed with autism by child’s race (See Additional file 2: Table S1). However, there were racial differences (of children and respondents) in the proportion of children screening positive on both the M-CHAT-R and M-CHAT-R/F, but not on the Q-CHAT-10 or Q-CHAT-10-O (See Additional file 2: Tables S2–S11). For the M-CHAT-R, M-CHAT-R/F and Q-CHAT-10, there were more positives among children with family incomes < $50,000; the Q-CHAT-10-O did not show this difference (See Additional file 2: Tables S1-S10).

Two one-sided tests for equivalence (TOST) were conducted to compare sensitivity, specificity, PPV and NPV between screening approaches [37, 38]. In all TOST analyses, a 5-point difference (*δ* = 0.05) in proportions was considered clinically significant. Each application of TOST utilizes two separate tests of proportions with *α* = 0.05 to arrive at one of four determinations (D: Relevant Difference [statistically & clinically significant], E: Equivalence [statistically & clinically significant], T: Trivial Difference [statistically significant, but clinically insignificant], I: Indeterminate Result [underpowered test for the data]).

The M-CHAT-R/F follow-up interview procedure increased PPV and specificity over the M-CHAT-R but decreased sensitivity (See Table 4; See also Additional file 3: Tables S4a–S4d for an alternative detailed view). Six of the 10 children with M-CHAT-R scores >  = 8 were diagnosed with autism. Since these high scores are unusual, we chose to explore this decision rule by performing a follow-up interview with these 10 even though the M-CHAT-R scoring indicates that they should be screened positive without relying on the interview. This follow-up interview for these 10 children was falsely negative for 3 of the 6 with an autism diagnosis. Q-CHAT-10 with standard scoring had higher PPV and specificity compared with both M-CHAT-R and M-CHAT-R/F. However, Q-CHAT-10 had lower sensitivity than M-CHAT-R.

**Table 4:** ASD screening performance comparisons

Row	Screen	Sensitivity	Specificity	PPV	NPV
1	M-CHAT-R *n* = 408	0.73 [0.61, 0.82] 2D | 3D | 4D	0.66 [0.61, 0.71] 2D | 3D | 4D	0.28 [0.22, 0.35] 2D | 3D | 4D	0.93 [0.89, 0.96] 2I | 3D | 4E
2	M-CHAT-R/F *n* = 368	0.36 [0.24, 0.49] 1D | 3I | 4D	0.89 [0.85, 0.92] 1D | 3D | 4D	0.36 [0.24, 0.49] 1D | 3D | 4I	0.89 [0.85, 0.92] 1I | 3E | 4I
3	Q-CHAT-10 *n* = 406	0.34 [0.23, 0.46] 1D | 2I | 4D	0.95 [0.92, 0.97] 1D | 2D | 4D	0.54 [0.39, 0.68] 1D | 2D | 4D	0.89 [0.85, 0.92] 1D | 2E | 4I
4	Q-CHAT-10-O *n* = 406	0.63 [0.50, 0.74] 1D | 2D | 3D	0.79 [0.74, 0.83] 1D | 2D | 3D	0.35 [0.27, 0.44] 1D | 2I | 3D	0.92 [0.89, 0.95] 1E | 2I | 3I

Another aim of this study was to explore the potential of ordinally scoring the Q-CHAT-10 by summing the full ordinal range of item responses rather than their dichotomized transformations. In this report ordinal re-scoring of the Q-CHAT-10 is denoted as Q-CHAT-10-O.

Ordinal scoring resulted in an area under the ROC curve (AUC) of 0.75 [0.71, 0.79] and a cutpoint of  >  = 12 that optimized the balance of sensitivity (0.63) versus specificity (0.79) via Youden’s J (0.42). Youden's J (J = sensitivity + specificity − 1) summarizes the performance of a dichotomous diagnostic test [39]. The index ranges from 0 to 1. A value of one indicates the test is perfect (no false positives or false negatives). In receiver operating characteristic (ROC) analyses, J is defined for each point on the ROC curve, and the maximum value of the index is used to select the optimum cutpoint when a test produces a numeric rather than dichotomous result [40]. The cutpoint >  = 12 was applied to Q-CHAT-10-O for comparisons of screening performance.

The M-CHAT-R/F is a two-stage procedure with different cutpoints for each stage. AUC analysis is not appropriate for M-CHAT-R/F given M-CHAT-R/F produces dichotomous results that are determined using discontinuous methods. However, it should be noted that the study’s chosen M-CHAT-R/F cutpoint of  >  = 2 is consistent with the recommended second stage cutpoint after verification of the parent’s responses following an M-CHAT-R screen positive based on a >  = 3 cutpoint.

Q-CHAT-10-O had higher specificity and PPV than M-CHAT-R with substantially less compromise in sensitivity than M-CHAT-R/F or Q-CHAT-10. However, Q-CHAT-10-O’s specificity was lower than M-CHAT-R/F and Q-CHAT-10.

Cronbach’s alpha for Q-CHAT-10 as a test scale was 0.55, and for Q-CHAT-10-O was 0.76. As a two-stage procedure the M-CHAT-R/F is not used in the conventional test theory manner of summing individual item responses/results. Therefore, internal consistency estimates are not appropriate and would be misleading.

## Discussion

When toddlers are screened in primary care at the 18-month visit, one cannot assume that children screening negative on the M-CHAT-R/F but positive on the M-CHAT-R or Q-CHAT screens are unlikely to have an autism diagnosis due to low the sensitivity of the M-CHAT-R/F. In fact, most children confirmed by diagnostic evaluations as having autism in this sample had negative M-CHAT-R/F follow-up interviews*.* Additionally, two community screening follow-up studies showed most children diagnosed with autism 2-1/2 to 7 years later had screened negative on the M-CHAT screen when 18 months old [24, 25]. However, as noted earlier, autism has been recognized as having a heterogenous trajectory of symptoms including many with the late onset of diagnosable symptoms [23, 24]*.* Unlike for typical clinical care when diagnostic testing is often delayed due to waiting lists or other issues, in this study we were often able to accommodate families through home testing and thereby completed all diagnostic testing prior to age two. The sensitivity estimates therefore should be more representative of children prior to the increases in prevalence expected by the natural history of this condition. The sensitivity estimate for the M-CHAT-R in this study was higher than in prior long-term outcome studies cited but lower than estimated in prior concurrent validation samples without screen negatives [15, 21–23]. This finding is in line with prospective data showing that most children diagnosed with autism at age three were not detectable at 18 months even with gold standard diagnostic testing [24]. It is also a response to recent commentary recommending that validity studies of autism screening tests focus on comparisons “against the gold standard assessment *at that target age.”* [41]

M-CHAT-R scoring without the follow-up was more sensitive to autism diagnoses than the recommended two-stage procedure (M-CHAT-R/F) but at the cost of lower PPV, consistent with prior M-CHAT-R studies [15]. Also consistent with prior studies at 18 months versus 24 months, inclusion of the follow-up interview still resulted in relatively low PPV [21, 22]. It should be noted that those M-CHAT-R/F estimates are limited by omission of the follow-up interview in some M-CHAT-R positive cases [21, 22]. Even with access to electronic support for completing the M-CHAT-R/F, the follow-up interview was inadvertently omitted 14% of the time in the current study and 59% of the time in a previous follow-up study when a similar application of electronic decision support was available [21]. On the other hand, studies of practices without any decision supports reveal that the follow-up interview is very rarely completed [17]. This study’s results are consistent with the recommendation to omit the follow-up interview in cases with M-CHAT-R scores >  = 8, rather than risking false negatives.

In a prior study, prediction of an autism diagnosis when pediatricians used online decision support for conducting the follow-up interview was equivalent to when used by autism center personnel [30]. That study also provided similar results at 24 months as in prior M-CHAT-R studies. This suggests that the differing results by age were not due to inaccurate follow-up interviews. There are a number of possible explanations for differing results across this age range that have potential implications for autism screening test development. In another prior study we found that when older toddlers (20 + months) were compared with younger (< 20 months), the younger toddlers had higher rates of item failure, with items that reflected more advanced developmental milestones having the highest failure rates [42], suggesting that autism screening tests may need age-related scoring cutpoints. Prospective studies suggest that autism symptoms emerge gradually [13], which may be reflected in a lower number of endorsed autism-specific items in younger children. These studies suggest that the early toddler age range studied here is dynamic in both the emergence of typical developmental milestones and the absence of them characterizing the autism syndrome. In the current context, “emergence” means the milestone may not be fully in place to be acknowledged by a yes or no response. Q-CHAT-10, with its ordinally scaled items, may better capture the nuanced manifestations of emerging developmental milestones.

Q-CHAT-10 with standard scoring showed greater specificity and PPV over M-CHAT-R and greater sensitivity than M-CHAT-R/F. While the Q-CHAT-10 is predicated on a “quantitative” range of responses, this study reveals that its simplified dichotomous scoring potentially compromises sensitivity relative to the ordinally scored Q-CHAT-10-O (0.47 vs. 0.66, respectively). While low resource settings might favor screens such as the Q-CHAT-10 with high specificity and low sensitivity, missing most of the affected individuals, this is not consistent with the public health goal of identifying all affected individuals in a population. Additional significant advantages of Q-CHAT-10-O over M-CHAT-R are that it requires half the number of items, and does not require a follow-up clinician interview. The Q-CHAT-10 and Q-CHAT-10-O, unlike the M-CHAT-R and M-CHAT-R/F, did not show significant overall differences in screen results across race of child or respondent. The lack of a significant difference across race was also true for autism diagnosis. The Q-CHAT-10-O was the only screen not showing a difference by family income. This may suggest that the format of graded responses and pictorial images may be less culturally biased. However, larger numbers of racial and economic subgroups are needed to confirm these impressions.

The limitations of the M-CHAT-R and its follow-up interview identified in this study are not an argument against the potential of parent-reported screening for autism in the 18-month well visit age group. Rather, they indicate a need for parent-reported tools that provide a more nuanced screening for emergent signs of autism in this age group. Simplified scoring of the M-CHAT-R with yes/no responses and of the Q-CHAT-10 with dichotomous cut points of ordinally scaled items runs counter to this goal. Such reductive techniques become less compelling as the availability of computing resources increases. Further, the simple sum score of Q-CHAT-10-O items may be as  easy as  the original scoring algorithm of Q-CHAT-10, and results in a measure with greater internal consistency.

This study represents one of the largest groups of toddlers with autism diagnostic testing before age two from a community sample. The sample of children could not feasibly include all children screened thus precluding an absolute estimate of screening sensitivity. We therefore presented the typical test performance estimates to allow comparison to other studies.

## Conclusions

This study reveals lower sensitivity to an autism diagnosis for the recommended two-stage M-CHAT-R/F than has been previously reported. There are two likely reasons for this discrepancy. First, this study differed from prior reports by obtaining diagnostic testing for children who screened negative on both autism screens as well as those who screened positive on at least one. Some of the screened negative children turned out to have autism diagnoses which would have been overlooked if we had not sampled them. Additionally, prior M-CHAT-R/F results have combined both 18- and 24-month well visit data, while our sample was exclusively collected at the 18-month visit. Prior studies have suggested less accuracy of screens at 18 months than at 24 months. Since the outcome comparison in this study involved timely completion of diagnostic testing, it provides a better estimate of what is possible when using these screens at the earliest currently recommended age for screening, occurring prior to some developmental shifts in the natural history of this condition [23–25]. The higher  sensitivity of the M-CHAT-R compared to M-CHAT-R/F occurred with less balancing benefit of increase in PPV as in studies including both 18- and 24-month visits. However, when the Q-CHAT-10 is scored using the full range of responses for each item (Q-CHAT-10-O), there are screening performance improvements over both the M-CHAT-R and M-CHAT-R/F. In addition, the Q-CHAT-10-O requires half the number of items, and has no requirement for a follow-up interview. The Q-CHAT-10-O is freely available from its authors and can be administered and scored without a computer. The Q-CHAT-10-O can therefore be recommended for autism screening at 18 months. As with all these parent-reported autism screens, this solution falls below generally accepted standards for screening performance [43] with a relatively low PPV, meaning most children screening positive will not be confirmed by diagnostic testing as having autism. While eliminating the follow-up interview is an important practical efficiency, it is possible that future research might identify follow-up questions for the Q-CHAT-10-O that might improve the limited positive predictive value which remains as a challenge at the 18-month visit. However, children with false positive screens for autism have been shown to have a high rate of “developmental concerns” [15]. When making an autism referral for young toddlers, clinicians might also consider the possibility that the child has a developmental problem other than autism and could thereby benefit from an evaluation even if the result is not an autism diagnosis. We intend to report separately on whether toddler autism screening can be enhanced by combining data from autism and developmental screeners. We will also explore possible clinical utility of false positive autism screen results for identifying other developmental problems as suggested by the M-CHAT-R authors [15]. Another strategy to consider is one of tracking and re-screening after 20 months, when M-CHAT-R/F screen results appear to be more accurate [19]. This approach may delay beginning important early intervention, however. Further research is needed for greater accuracy of screening at the 18-month visit, possibly including greater sensitivity to language outcomes not seen in any of the screens reported here. Our group is developing a screening solution involving promising parent report adaptive computer-based strategies utilizing language items and more fully integrating autism screening with screening for developmental delay [19, 44]. In a separate study we have shown that no screen at any age group identifies all or even most autism cases suggesting that autism screening should be conducted continuously at different ages during childhood, adolescence, and adulthood [45]. Digital behavioral measures, e.g., visual gaze, may also hold promise when and if they become practical and validated in primary care settings [46].

## Limitations

The sample in this study is not fully representative of the population of interest. Many contacted parents declined participation, more so in the screened negative group than in the screened positive group; parent participants were more highly educated than national rates; and children exposed to English less than fifty percent of the time were excluded. The Q-CHAT-10-O derives its score from the ordinal responses to the standard Q-CHAT-10 used in this study. It is possible that if the Q-CHAT-10-O had been used as the screen for the primary sample, some children may have screened positive and been more likely to have been recruited to the final sample. It should be noted that autism diagnoses were made without a standard caregiver report tool such as the Autism Diagnostic Interview-Revised (ADI-R) Algorithms for Toddlers and Young Preschoolers based on opinion that such data tends to be less accurate in young toddler samples identified by screening rather than parent concern [47]. However, the diagnostic results may have been different had such a tool been used. The version of the Q-CHAT-10 used in this study included illustrations of items as suggested by the authors, with unknown impact compared to the original. Results from these studies of 16–20-month-olds cannot be extended to older ages.

## Supplementary Information

**Additional file 1**. Supplementary tables: Outcomes by Screening Results.

**Additional file 2**. Supplementary tables: Patient & Respondent Demographics by Screening Result.

**Additional file 3**. Supplementary tables: Autism Screening Performance Comparisons.

## Data Availability

The dataset used and analyzed during the current study is available from the corresponding author on reasonable request. Data is available from the corresponding author for reasonable requests.

## Abbreviations

PCP: Primary care provider
M-CHAT-R: Modified checklist for Autism in Toddlers-Revised
M-CHAT-R/F: Modified Checklist for Autism in Toddlers Follow-up Interview
ADOS-2: The Autism Diagnostic Observation Schedule, Second Edition
MSEL: Mullen Scales of Early Learning
ROC: Receiver operating characteristics
Q-CHAT-10: Quantitative Checklist for Autism in Toddlers-10 item version
Q-CHAT-10-O: Q-CHAT-10 scored through full range of ordinal items
AUC: Area under the curve
CHADIS: Comprehensive Health And Decision Information System
PPV: Positive predictive value
AAP: American Academy of Pediatrics
CDC: Centers for Disease Control and Prevention
