# Supplementary material for: Autism screening at 18 months of age: a comparison of the Q-CHAT-10 and M-CHAT screeners
Source: Mol Autism. 2022 Jan 3;13:2. doi: 10.1186/s13229-021-00480-4 (PMC8722322; doi:10.1186/s13229-021-00480-4)
Supplement: Supplementary file 1 — Additional file 1. Supplementary tables: Outcomes by Screening Results. [file 13229_2021_480_MOESM1_ESM.pdf]

Table 1 - Developmental Diagnostic Outcomes by Autism Diagnosis

	Autism Negative	Autism Positive	Difference	*t*	df	*p*
Gross Motor T-Score	51.65	46.02	-5.63	4.330	403	< .001
Fine Motor T-Score	51.76	44.13	-7.62	6.231	403	< .001
Visual Reception T-Score	56.29	44.33	-11.97	7.680	402	< .001
Receptive Language T-Score	53.62	31.82	-21.80	11.142	402	< .001
Expressive Language T-Score	48.22	33.05	-15.17	8.710	403	< .001

Table 2 - Developmental Diagnostic Outcomes by M-CHAT-R Result

	M-CHAT-R Negative	M-CHAT-R Positive	Difference	*t*	df	*p*
Gross Motor T-Score	52.33	48.52	-3.82	4.016	403	< .001
Fine Motor T-Score	52.08	48.42	-3.67	4.000	403	< .001
Visual Reception T-Score	57.13	50.58	-6.56	5.589	402	< .001
Receptive Language T-Score	55.32	42.95	-12.37	8.176	402	< .001
Expressive Language T-Score	49.93	40.00	-9.93	7.680	403	< .001

Table 3 - Developmental Diagnostic Outcomes by M-CHAT-R/F Result

	M-CHAT-R/F Negative	M-CHAT-R/F Positive	Difference	*t*	df	*p*
Gross Motor T-Score	51.78	46.34	-5.44	3.984	363	< .001
Fine Motor T-Score	52.02	44.70	-7.32	5.659	363	< .001
Visual Reception T-Score	56.40	47.47	-8.93	5.248	362	< .001
Receptive Language T-Score	53.67	39.28	-14.39	6.546	362	< .001
Expressive Language T-Score	48.37	37.66	-10.71	5.497	363	< .001

Table 4 - Developmental Diagnostic Outcomes by Q-CHAT-10 Result

	Q-CHAT-10 Negative	Q-CHAT-10 Positive	Difference	*t*	df	*p*
Gross Motor T-Score	51.48	44.47	-7.01	4.387	401	< .001
Fine Motor T-Score	51.39	43.11	-8.28	5.446	401	< .001
Visual Reception T-Score	55.60	44.05	-11.54	5.870	400	< .001
Receptive Language T-Score	52.12	34.42	-17.70	6.831	400	< .001
Expressive Language T-Score	47.31	33.29	-14.02	6.311	401	< .001

Table 5 - Developmental Diagnostic Outcomes by Positive on M-CHAT-R OR Q-CHAT-10

	No	Yes	Difference	*t*	df	*p*
Gross Motor T-Score	52.32	48.63	-3.69	3.869	401	< .001
Fine Motor T-Score	52.13	48.38	-3.75	4.078	401	< .001
Visual Reception T-Score	57.18	50.62	-6.56	5.581	400	< .001
Receptive Language T-Score	55.47	43.16	-12.30	8.150	400	< .001
Expressive Language T-Score	50.15	39.93	-10.22	7.935	401	< .001

Table 6 - Autism Diagnosis by Screening Result

	Screen Negative	Screen Positive	Difference	N	*z*	*p*
Autism Positive | M-CHAT-R Result	6.97%	28.05%	-21.08%	408	-5.78	< .001
Autism Positive | M-CHAT-R/F Result	10.79%	35.85%	-25.06%	368	-4.81	< .001
Autism Positive | Q-CHAT-10 Result	11.17%	53.85%	-42.67%	406	-7.04	< .001
Autism Positive | (M-CHAT-R or Q-CHAT-10) Result	6.64%	27.88%	-21.24%	406	-5.84	< .001
