# Supplementary material for: Autism screening at 18 months of age: a comparison of the Q-CHAT-10 and M-CHAT screeners
Source: Mol Autism. 2022 Jan 3;13:2. doi: 10.1186/s13229-021-00480-4 (PMC8722322; doi:10.1186/s13229-021-00480-4)
Supplement: Supplementary file 2 — Additional file 2. Supplementary tables: Patient & Respondent Demographics by Screening Result. [file 13229_2021_480_MOESM2_ESM.pdf]

Table 1 - Patient Demographics by Autism Diagnosis

	Autism Diagnosis					
	Negative		Positive			
	n	%	n	%	χ	*p*
Diagnosis Age (Months) - [Mean (SD)]	20.52	(1.87)	20.29	(1.80)	*t* = 0.86	.389
Diagnosis Age (Months) - [Min Max]	16.76	31.54	16.99	26.71		
Sex					1.21	.271
Female	100	28.99%	14	22.22%		
Male	245	71.01%	49	77.78%		
Race					8.16	.086
Asian	15	4.39%	4	6.56%		
Black	28	8.19%	11	18.03%		
White	245	71.64%	36	59.02%		
Multiple	48	14.04%	10	16.39%		
Unknown	6	1.75%	0	0.00%		
Hispanic or Latino					0.43	.806
No	319	93.27%	56	91.80%		
Yes	22	6.43%	5	8.20%		
Private Payer					6.46	.011
Yes	300	87.72%	46	75.41%		
Public Payer					3.81	.051
Yes	36	10.43%	12	19.05%		
Other Payer					0.29	.593
Yes	41	11.88%	9	14.29%		
Household Income					19.88	< .001
< $50,000	9	3.85%	9	22.50%		
$50,000 - $150,000	114	48.72%	18	45.00%		
>= $150,000	111	47.44%	13	32.50%		

Table 2 - Patient Demographics by MCHAT-R Result

	MCHAT-R Result					
	Negative		Positive			
	n	%	n	%	χ	*p*
Diagnosis Age (Months) - [Mean (SD)]	18.02	(0.45)	18.01	(0.64)	*t = 0.22*	.822
Diagnosis Age (Months) - [Min Max]	16.13	19.32	16.03	19.91		
Sex					1.93	.165
Female	62	25.41%	52	31.71%		
Male	182	74.59%	112	68.29%		
Race					16.21	.003
Asian	11	4.55%	8	4.97%		
Black	14	5.79%	25	15.53%		
White	180	74.38%	101	62.73%		
Multiple	36	14.88%	22	13.66%		
Unknown	1	0.41%	5	3.11%		
Hispanic or Latino					1.52	.468
No	226	93.39%	149	92.55%		
Yes	16	6.61%	11	6.83%		
Private Payer					4.45	.035
Yes	215	88.84%	131	81.37%		
Public Payer					5.83	.016
Yes	21	8.61%	27	16.46%		
Other Payer					0.08	.781
Yes	29	11.89%	21	12.80%		
Household Income					13.48	.001
< $50,000	5	2.91%	13	12.75%		
$50,000 - $150,000	79	45.93%	53	51.96%		
>= $150,000	88	51.16%	36	35.29%		

Table 3 - Patient Demographics by MCHAT-R/F Result

	MCHAT-R/F Result					
	Negative		Positive			
	n	%	n	%	χ	*p*
Diagnosis Age (Months) - [Mean (SD)]	18.02	(0.47)	17.98	(0.74)	*t* = 0.53	.594
Diagnosis Age (Months) - [Min Max]	16.10	19.48	16.03	19.91		
Sex					0.37	.544
Female	90	28.57%	13	24.53%		
Male	225	71.43%	40	75.47%		
Race					31.39	< .001
Asian	16	5.11%	2	3.85%		
Black	23	7.35%	10	19.23%		
White	226	72.20%	33	63.46%		
Multiple	47	15.02%	4	7.69%		
Unknown	1	0.32%	3	5.77%		
Hispanic or Latino					1.28	.527
No	294	93.93%	47	90.38%		
Yes	18	5.75%	5	9.62%		
Private Payer					2.60	.107
Yes	273	87.22%	41	78.85%		
Public Payer					7.20	.007
Yes	31	9.84%	12	22.64%		
Other Payer					0.03	.866
Yes	39	12.38%	7	13.21%		
Household Income					16.76	< .00
< $50,000	9	4.05%	7	23.33%		
$50,000 - $150,000	108	48.65%	13	43.33%		
>= $150,000	105	47.30%	10	33.33%		

Table 4 - Patient Demographics by QCHAT-10 Result

	QCHAT-10 Result					
	Negative		Positive			
	n	%	n	%	χ	*p*
Diagnosis Age (Months) - [Mean (SD)]	18.01	(0.53)	18.08	(0.60)	*t* = -0.83	.406
Diagnosis Age (Months) - [Min Max]	16.03	19.91	16.23	19.32		
Sex					6.64	.010
Female	109	29.70%	4	10.26%		
Male	258	70.30%	35	89.74%		
Race					7.88	.096
Asian	19	5.23%	0	0.00%		
Black	32	8.82%	6	15.79%		
White	255	70.25%	25	65.79%		
Multiple	53	14.60%	5	13.16%		
Unknown	4	1.10%	2	5.26%		
Hispanic or Latino					1.14	.565
No	337	92.84%	37	97.37%		
Yes	25	6.89%	1	2.63%		
Private Payer					7.84	.005
Yes	318	87.60%	27	71.05%		
Public Payer					3.62	.057
Yes	38	10.35%	8	20.51%		
Other Payer					0.38	.540
Yes	44	11.99%	6	15.38%		
Household Income					7.42	.025
< $50,000	13	5.18%	4	18.18%		
$50,000 - $150,000	120	47.81%	12	54.55%		
>= $150,000	118	47.01%	6	27.27%		

Table 5 - Patient Demographics by QCHAT-10-O Result

	QCHAT-10-O Result					
	Negative		Positive			
	n	%	n	%	χ	*p*
Diagnosis Age (Months) - [Mean (SD)]	18.00	(0.52)	18.06	(0.57)	*t* = -0.96	.340
Diagnosis Age (Months) - [Min Max]	16.03	19.91	16.07	19.32		
Sex					2.93	.087
Female	89	30.17%	24	21.62%		
Male	206	69.83%	87	78.38%		
Race					2.13	.712
Asian	13	4.47%	6	5.45%		
Black	25	8.59%	13	11.82%		
White	209	71.82%	71	64.55%		
Multiple	40	13.75%	18	16.36%		
Unknown	4	1.37%	2	1.82%		
Hispanic or Latino					2.65	.265
No	272	93.47%	102	92.73%		
Yes	19	6.53%	7	6.36%		
Private Payer					3.31	.069
Yes	256	87.97%	89	80.91%		
Public Payer					0.72	.395
Yes	31	10.51%	15	13.51%		
Other Payer					0.01	.911
Yes	36	12.20%	14	12.61%		
Household Income					4.70	.095
< $50,000	9	4.43%	8	11.43%		
$50,000 - $150,000	98	48.28%	34	48.57%		
>= $150,000	96	47.29%	28	40.00%		

Table 6 - Patient Demographics by Positive on M-CHAT-R OR Q-CHAT-10

	M-CHAT-R OR Q-CHAT-10					
	Negative		Positive			
	n	%	n	%	χ	*p*
Diagnosis Age (Months) - [Mean (SD)]	18.03	(0.44)	18.00	(0.65)	*t* = 0.52	.606
Diagnosis Age (Months) - [Min Max]	16.13	19.32	16.03	19.91		
Sex					1.31	.252
Female	62	25.73%	51	30.91%		
Male	179	74.27%	114	69.09%		
Race					14.64	.006
Asian	11	4.60%	8	4.94%		
Black	14	5.86%	24	14.81%		
White	178	74.48%	102	62.96%		
Multiple	35	14.64%	23	14.20%		
Unknown	1	0.42%	5	3.09%		
Hispanic or Latino					1.52	.469
No	223	93.31%	151	93.21%		
Yes	16	6.69%	10	6.17%		
Private Payer					4.69	.030
Yes	213	89.12%	132	81.48%		
Public Payer					4.04	.044
Yes	21	8.71%	25	15.15%		
Other Payer					0.27	.605
Yes	28	11.62%	22	13.33%		
Household Income					12.41	.002
< $50,000	5	2.92%	12	11.76%		
$50,000 - $150,000	78	45.61%	54	52.94%		
>= $150,000	88	51.46%	36	35.29%		

Table 7 - Respondent Demographics by Austism Diagnosis

	Autism Diagnosis					
	Negative		Positive			
	n	%	n	%	χ	*p*
Relationship to Child					0.89	.641
Mother	316	91.59%	58	92.06%		
Father	27	7.83%	4	6.35%		
Other Primary	2	0.58%	1	1.59%		
Age - [Mean (SD)]	34.32	(4.26)	33.98	(4.77)	*t* = 0.55	.585
Age - [Min Max]	19	46	24	43		
Marital Status					13.81	.017
Married	319	93.27%	49	80.33%		
Separated	2	0.58%	2	3.28%		
Widowed	1	0.29%	0	0.00%		
Never married	7	2.05%	5	8.20%		
Living with partner	11	3.22%	4	6.56%		
Race					11.65	.020
Asian	22	6.38%	7	11.11%		
Black	25	7.25%	12	19.05%		
White	274	79.42%	40	63.49%		
Multiple	18	5.22%	3	4.76%		
Unknown	6	1.74%	1	1.59%		
Hispanic or Latino					0.26	.876
No	321	93.86%	58	95.08%		
Yes	20	5.85%	3	4.92%		
Bachelor Degree					4.07	.044
No	58	16.96%	17	27.87%		
Yes	284	83.04%	44	72.13%		
Household Income					19.88	< .001
< $50,000	9	3.85%	9	22.50%		
$50,000 - $150,000	114	48.72%	18	45.00%		
>= $150,000	111	47.44%	13	32.50%		

Table 8 - Respondent Demographics by MCHAT-R Result

	MCHAT-R Result					
	Negative		Positive			
	n	%	n	%	χ	*p*
Relationship to Child					0.40	.820
Mother	225	92.21%	149	90.85%		
Father	17	6.97%	14	8.54%		
Other Primary	2	0.82%	1	0.61%		
Age - [Mean (SD)]	34.31	(4.05)	34.20	(4.76)	$t = 0.24$	.810
Age - [Min Max]	24	43	19	46		
Marital Status					8.64	.124
Married	226	93.39%	142	88.20%		
Separated	0	0.00%	4	2.48%		
Widowed	1	0.41%	0	0.00%		
Never married	6	2.48%	6	3.73%		
Living with partner	7	2.89%	8	4.97%		
Race					16.60	.002
Asian	15	6.15%	14	8.54%		
Black	14	5.74%	23	14.02%		
White	201	82.38%	113	68.90%		
Multiple	13	5.33%	8	4.88%		
Unknown	1	0.41%	6	3.66%		
Hispanic or Latino					1.76	.414
No	227	93.80%	152	94.41%		
Yes	15	6.20%	8	4.97%		
Bachelor Degree					13.46	< .001
No	31	12.81%	44	27.33%		
Yes	211	87.19%	117	72.67%		
Household Income					13.48	.001
< $50,000	5	2.91%	13	12.75%		
$50,000 - $150,000	79	45.93%	53	51.96%		
>= $150,000	88	51.16%	36	35.29%		

Table 9 - Respondent Demographics by MCHAT-R/F Result

	MCHAT-R/F Result					
	Negative		Positive			
	n	%	n	%	χ	*p*
Relationship to Child					0.70	.705
Mother	288	91.43%	48	90.57%		
Father	24	7.62%	5	9.43%		
Other Primary	3	0.95%	0	0.00%		
Age - [Mean (SD)]	34.27	(4.13)	34.08	(5.36)	$t = 0.28$	.777
Age - [Min Max]	24	45	19	46		
Marital Status					19.76	.001
Married	292	93.29%	44	84.62%		
Separated	0	0.00%	3	5.77%		
Widowed	1	0.32%	0	0.00%		
Never married	9	2.88%	2	3.85%		
Living with partner	9	2.88%	2	3.85%		
Race					23.78	< .001
Asian	22	6.98%	3	5.66%		
Black	23	7.30%	9	16.98%		
White	254	80.63%	35	66.04%		
Multiple	15	4.76%	2	3.77%		
Unknown	1	0.32%	4	7.55%		
Hispanic or Latino					0.21	.903
No	296	94.57%	49	94.23%		
Yes	16	5.11%	3	5.77%		
Bachelor Degree					18.37	< .001
No	44	14.06%	20	38.46%		
Yes	269	85.94%	32	61.54%		
Household Income					16.76	< .001
< $50,000	9	4.05%	7	23.33%		
$50,000 - $150,000	108	48.65%	13	43.33%		
>= $150,000	105	47.30%	10	33.33%		

Table 10 - Respondent Demographics by QCHAT-10 Result

	QCHAT-10 Result					
	Negative		Positive			
	n	%	n	%	χ	*p*
Relationship to Child					1.96	.375
Mother	337	91.83%	35	89.74%		
Father	28	7.63%	3	7.69%		
Other Primary	2	0.54%	1	2.56%		
Age - [Mean (SD)]	34.27	(4.20)	34.24	(5.61)	*t* = 0.05	.961
Age - [Min Max]	24	46	19	43		
Marital Status					15.70	.008
Married	337	92.84%	29	76.32%		
Separated	3	0.83%	1	2.63%		
Widowed	1	0.28%	0	0.00%		
Never married	8	2.20%	4	10.53%		
Living with partner	11	3.03%	4	10.53%		
Race					6.01	0.198
Asian	28	7.63%	1	2.56%		
Black	30	8.17%	6	15.38%		
White	286	77.93%	27	69.23%		
Multiple	17	4.63%	4	10.26%		
Unknown	6	1.63%	1	2.56%		
Hispanic or Latino					2.67	.263
No	339	93.39%	38	100.00 %		
Yes	23	6.34%	0	0.00%		
Bachelor Degree					6.93	.008
No	61	16.80%	13	34.21%		
Yes	302	83.20%	25	65.79%		
Household Income					7.42	.025
< $50,000	13	5.18%	4	18.18%		
$50,000 - $150,000	120	47.81%	12	54.55%		
>= $150,000	118	47.01%	6	27.27%		

Table 11 - Respondent Demographics by QCHAT-10-O Result

	QCHAT-10-O Result					
	Negative		Positive			
	n	%	n	%	χ	*p*
Relationship to Child					1.19	.552
Mother	273	92.54%	99	89.19%		
Father	20	6.78%	11	9.91%		
Other Primary	2	0.68%	1	0.90%		
Age - [Mean (SD)]	34.32	(4.06)	34.13	(5.06)	*t* = 0.39	.696
Age - [Min Max]	24	46	19	45		
Marital Status					13.23	.021
Married	274	94.16%	92	83.64%		
Separated	2	0.69%	2	1.82%		
Widowed	0	0.00%	1	0.91%		
Never married	5	1.72%	7	6.36%		
Living with partner	8	2.75%	7	6.36%		
Race					6.35	.174
Asian	17	5.76%	12	10.81%		
Black	22	7.46%	14	12.61%		
White	235	79.66%	78	70.27%		
Multiple	16	5.42%	5	4.50%		
Unknown	5	1.69%	2	1.80%		
Hispanic or Latino					2.67	.263
No	274	94.16%	103	93.64%		
Yes	17	5.84%	6	5.45%		
Bachelor Degree					3.74	.053
No	47	16.15%	27	24.55%		
Yes	244	83.85%	83	75.45%		
Household Income					4.70	.095
< $50,000	9	4.43%	8	11.43%		
$50,000 - $150,000	98	48.28%	34	48.57%		
>= $150,000	96	47.29%	28	40.00%		

Table 12 - Respondent Demographics by Positive on M-CHAT-R OR Q-CHAT-10

	M-CHAT-R OR Q-CHAT-10					
	Negative		Positive			
	n	%	n	%	χ	*p*
Relationship to Child					0.34	.842
Mother	222	92.12%	150	90.91%		
Father	17	7.05%	14	8.48%		
Other Primary	2	0.83%	1	0.61%		
Age - [Mean (SD)]	34.36	(4.01)	34.13	(4.82)	$t = 0.52$	.603
Age - [Min Max]	24	43	19	46		
Marital Status					8.41	.135
Married	223	93.31%	143	88.27%		
Separated	0	0.00%	4	2.47%		
Widowed	1	0.42%	0	0.00%		
Never married	6	2.51%	6	3.70%		
Living with partner	7	2.93%	8	4.94%		
Race					15.20	.004
Asian	15	6.22%	14	8.48%		
Black	14	5.81%	22	13.33%		
White	199	82.57%	114	69.09%		
Multiple	12	4.98%	9	5.45%		
Unknown	1	0.41%	6	3.64%		
Hispanic or Latino					1.78	.410
No	224	93.72%	153	94.44%		
Yes	15	6.28%	8	4.94%		
Bachelor Degree					13.69	< .001
No	30	12.55%	44	27.16%		
Yes	209	87.45%	118	72.84%		
Household Income					12.41	.002
< $50,000	5	2.92%	12	11.76%		
$50,000 - $150,000	78	45.61%	54	52.94%		
>= $150,000	88	51.46%	36	35.29%		
