# Supplementary material for: Autism screening at 18 months of age: a comparison of the Q-CHAT-10 and M-CHAT screeners
Source: Mol Autism. 2022 Jan 3;13:2. doi: 10.1186/s13229-021-00480-4 (PMC8722322; doi:10.1186/s13229-021-00480-4)
Supplement: Supplementary file 3 — Additional file 3. Supplementary tables: Autism Screening Performance Comparisons. [file 13229_2021_480_MOESM3_ESM.pdf]

Table 4a - ASD Screening Performance Comparisons - Comparand: M-CHAT-R

Screen	Sensitivity	Specificity	PPV	NPV
**M-CHAT-R n=408**	**0.73 [0.61, 0.82]**	**0.66 [0.61, 0.71]**	**0.28 [0.22, 0.35]**	**0.93 [0.89, 0.96]**
M-CHAT-R/F n=368	0.36 [0.24, 0.49] Difference	0.89 [0.85, 0.92] Difference	0.36 [0.24, 0.49] Difference	0.89 [0.85, 0.92] Indeterminate
Q-CHAT-10 n=406	0.34 [0.23, 0.46] Difference	0.95 [0.92, 0.97] Difference	0.54 [0.39, 0.68] Difference	0.89 [0.85, 0.92] Difference
Q-CHAT-10-O n=406	0.63 [0.50, 0.74] Difference	0.79 [0.74, 0.83] Difference	0.35 [0.27, 0.44] Difference	0.92 [0.89, 0.95] Equivalence

Table 4b - ASD Screening Performance Comparisons - Comparand: M-CHAT-R/F

Screen	Sensitivity	Specificity	PPV	NPV
M-CHAT-R n=408	0.73 [0.61, 0.82] Difference	0.66 [0.61, 0.71] Difference	0.28 [0.22, 0.35] Difference	0.93 [0.89, 0.96] Indeterminate
**M-CHAT-R/F n=368**	**0.36 [0.24, 0.49]**	**0.89 [0.85, 0.92]**	**0.36 [0.24, 0.49]**	**0.89 [0.85, 0.92]**
Q-CHAT-10 n=406	0.34 [0.23, 0.46] Indeterminate	0.95 [0.92, 0.97] Difference	0.54 [0.39, 0.68] Difference	0.89 [0.85, 0.92] Equivalence
Q-CHAT-10-O n=406	0.63 [0.50, 0.74] Difference	0.79 [0.74, 0.83] Difference	0.35 [0.27, 0.44] Indeterminate	0.92 [0.89, 0.95] Indeterminate

Table 4c - ASD Screening Performance Comparisons - Comparand: Q-CHAT-10

Screen	Sensitivity	Specificity	PPV	NPV
M-CHAT-R n=408	0.73 [0.61, 0.82] Difference	0.66 [0.61, 0.71] Difference	0.28 [0.22, 0.35] Difference	0.93 [0.89, 0.96] Difference
M-CHAT-R/F n=368	0.36 [0.24, 0.49] Indeterminate	0.89 [0.85, 0.92] Difference	0.36 [0.24, 0.49] Difference	0.89 [0.85, 0.92] Equivalence
**Q-CHAT-10 n=406**	**0.34 [0.23, 0.46]**	**0.95 [0.92, 0.97]**	**0.54 [0.39, 0.68]**	**0.89 [0.85, 0.92]**
Q-CHAT-10-O n=406	0.63 [0.50, 0.74] Difference	0.79 [0.74, 0.83] Difference	0.35 [0.27, 0.44] Difference	0.92 [0.89, 0.95] Indeterminate

Table 4d - ASD Screening Performance Comparisons - Comparand: Q-CHAT-10-O

Screen	Sensitivity	Specificity	PPV	NPV
M-CHAT-R n=408	0.73 [0.61, 0.82] Difference	0.66 [0.61, 0.71] Difference	0.28 [0.22, 0.35] Difference	0.93 [0.89, 0.96] Equivalence
M-CHAT-R/F n=368	0.36 [0.24, 0.49] Difference	0.89 [0.85, 0.92] Difference	0.36 [0.24, 0.49] Indeterminate	0.89 [0.85, 0.92] Indeterminate
Q-CHAT-10 n=406	0.34 [0.23, 0.46] Difference	0.95 [0.92, 0.97] Difference	0.54 [0.39, 0.68] Difference	0.89 [0.85, 0.92] Indeterminate
**Q-CHAT-10-O n=406**	**0.63 [0.50, 0.74]**	**0.79 [0.74, 0.83]**	**0.35 [0.27, 0.44]**	**0.92 [0.89, 0.95]**
